# Segmental duplications are hot spots of copy number variants affecting barley gene content

**DOI:** 10.1111/tpj.14784

**Published:** 2020-05-17

**Authors:** Gianluca Bretani, Laura Rossini, Chiara Ferrandi, Joanne Russell, Robbie Waugh, Benjamin Kilian, Paolo Bagnaresi, Luigi Cattivelli, Agostino Fricano

**Affiliations:** ^1^ Università degli Studi di Milano – DiSAA Via Celoria 2 20133 Milano Italy; ^2^ Parco Tecnologico Padano Loc. C.na Codazza Via Einstein 26900 Lodi Italy; ^3^ James Hutton Institute, Invergowrie Dundee DD2 5DA UK; ^4^ Leibniz Institute of Plant Genetics and Crop Plant Research (IPK) Corrensstrasse 3 06466 Gatersleben Germany; ^5^ Council for Agricultural Research and Economics – Research Centre for Genomics & Bioinformatics Via San Protaso 302 29017 Fiorenzuola d'Arda (PC) Italy; ^6^ Global Crop Diversity Trust Platz der Vereinten Nationen 7 53113 Bonn Germany

**Keywords:** barley, copy number variants, segmental duplications, exome sequencing

## Abstract

Copy number variants (CNVs) are pervasive in several animal and plant genomes and contribute to shaping genetic diversity. In barley, there is evidence that changes in gene copy number underlie important agronomic traits. The recently released reference sequence of barley represents a valuable genomic resource for unveiling the incidence of CNVs that affect gene content and for identifying sequence features associated with CNV formation. Using exome sequencing and read count data, we detected 16 605 deletions and duplications that affect barley gene content by surveying a diverse panel of 172 cultivars, 171 landraces, 22 wild relatives and other 32 uncategorized domesticated accessions. The quest for segmental duplications (SDs) in the reference sequence revealed many low‐copy repeats, most of which overlap predicted coding sequences. Statistical analyses revealed that the incidence of CNVs increases significantly in SD‐rich regions, indicating that these sequence elements act as hot spots for the formation of CNVs. The present study delivers a comprehensive genome‐wide study of CNVs affecting barley gene content and implicates SDs in the molecular mechanisms that lead to the formation of this class of CNVs.

## Significance Statement

In the present study, we examined the diversity and distribution of CNVs that affect gene content in 397 diverse barley accessions. Leveraging the reference sequence of the barley cultivar ‘Morex’, we show that CNVs occur preferentially in SD‐rich regions.

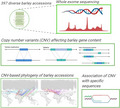

## INTRODUCTION

Copy number variants (CNVs) are a class of unbalanced structural changes within genomes, which represent either a gain of extra sequence copies (duplications or insertions), or a loss of genetic material (deletions) in individuals of the same species (Alkan *et al*., 2011). In the human genome, CNVs were generally defined as deletions, insertions and duplications of DNA sequences longer than 1 kb (Feuk *et al*., 2006), although small structural changes of 50 bp or larger are now also considered as CNVs (Alkan *et al*., 2011; Girirajan *et al*., 2011).

Although several studies in plants have analysed genomic variability in terms of single nucleotide polymorphisms (SNPs), investigations of the CNV rate, diversity and impact on genomic variation are lagging behind. For example, years of empirical breeding and selection of crops narrowed the number of SNP variants in the cultivated gene pool (Kilian *et al.*, 2007; Fricano *et al.*, 2009), although it is still unclear whether this process might also have eroded CNV diversity. In barley, the contribution of CNVs in shaping genetic diversity is largely unknown: to date, systematic analyses for identifying short CNVs have been carried out on a very limited panel of domesticated and wild accessions using a gene‐space assembly (International Barley Genome Sequencing Consortium *et al*., 2012; Muñoz‐Amatriaín *et al.*, 2013).

Genome‐wide surveys leading to the discovery of thousands of CNVs have revealed a ubiquity of deletions and duplications in maize, tale cress, rice and switchgrass (Springer *et al.*, 2009; Debolt, 2010; Swanson‐wagner *et al.*, 2010; Evans *et al.*, 2015; Bai *et al.*, 2016). Beyond affecting genome structure, CNVs have the potential to modulate or create new gene functions. There is evidence that CNVs, along with other structural variants, play key roles in plant adaptive evolution, as well as in human diseases (Freeman *et al*., 2006; Kim *et al.*, 2008; Evans *et al.*, 2015; Pinosio *et al.*, 2016; Prunier *et al.*, 2017). In both plant and animal kingdoms, genes exhibiting CNVs are related to defense, biotic and abiotic stress responses (Conrad *et al.*, 2010; Clop *et al*., 2012; Pinosio *et al.*, 2016; Prunier *et al.*, 2017). In barley, the genetic dissection of boron‐toxicity tolerance demonstrated that duplications of *Bot1* underlie this trait (Sutton *et al.*, 2007), whereas duplications of *HvFT1* are tied to earlier flowering and have an overriding effect on the vernalization mechanism (Loscos *et al.*, 2014). In wheat, duplications of *Vrn‐1A* and *Ppd‐1B* were demonstrated to affect vernalization requirement and the photoperiod response, respectively (Díaz *et al.*, 2012). Apart from these notable examples, the incidence and the functions of genes exhibiting CNVs are still unknown.

Segmental duplications (SDs) (also termed ‘low‐copy repeats’) are stretches of high complexity DNA sequences longer than 1 kb, which are repeated several times in the genome with nucleotide identity higher than 90% (Eichler, 2001). Genome analyses and the creation of high quality reference sequences of plant and animal species have shown that SDs are common elements of genomes (Pagel *et al.*, 2004; Sharp *et al.*, 2005; Innan and Kondrashov, 2010; Giannuzzi *et al.*, 2011; Zhang *et al.*, 2017). In barley, annotation of the reference sequence revealed that more than 75% of genes belong to families with multiple members, suggesting that duplications of DNA sequences contributed to shaping both gene content and function (Mascher *et al.*, 2017). For example, the reference sequence of barley cultivar (cv) ‘Morex’ contains five complete genes of *amy1* family, four of which share more than 99.8% nucleotide identity, computed considering intron and exon sequences (Mascher *et al.*, 2017). The abundance of gene families with multiple members hints that low‐copy repeats could extend beyond the coding portion of the barley genome and play a fundamental role in shaping CNVs.

Several mammalian genome studies showed that SDs are hotspots of genome instability because they predispose chromosomes to rearrangements, providing templates for non‐allelic homologous recombination (NAHR) events (Sharp *et al.*, 2005; Kim *et al.*, 2008; Dittwald *et al.*, 2013; Zhang *et al.*, 2015). Based on the distribution of SDs in the human genome, it was suggested that recent SDs could play a role in the formation of specific classes of CNVs via NAHR (Sharp *et al.*, 2005; Freeman *et al*., 2006; Hastings *et al*., 2010). Beyond this mechanism, other types of processes that lead to CNV formation have been proposed, including non‐homologous DNA repair (Hastings *et al*., 2010). This class of molecular mechanisms includes non‐homologous end‐joining, breakage micro‐homology‐mediated end‐joining, template switching as a result of fork stalling or replication slippage and micro‐homology‐mediated break‐induced replication (Hastings *et al*., 2010). In barley, a portion of short CNVs has a sequence signature of being formed by non‐homologous DNA repair (Muñoz‐Amatriaín *et al.*, 2013), although the mechanisms that generate longer CNVs are still unknown.

In the present study, we examined the diversity and distribution of CNVs that affect barley gene content. We used exome capture sequencing data from a panel of 397 diverse barley accessions to assess the occurrence and distribution of CNVs across the barley genome. Leveraging the newly created reference sequence of the barley cv ‘Morex’ (Mascher *et al.*, 2017), we show that CNVs occur preferentially in SD‐rich regions.

## RESULTS

### Identification and distribution of CNVs affecting barley gene content

To identify the genome‐wide occurrence of gene duplications and deletions, we employed a detection strategy based on exome capture sequencing of a panel of 397 (of 403) diverse accessions that have been described previously (Bustos‐Korts *et al.*, 2019), including 172 cultivars, 171 landraces, 22 wild relatives and other 32 domesticated accessions for which the categorization as cultivar or landrace was questionable (Table S1). Target regions used to design the exome capture probes were mapped to the reference sequence of barley cv ‘Morex’ (Mascher *et al.*, 2017), which allowed us to establish that the target space covers 170 725 exons or sequence intervals. Overall, the captured sequences encompass 61.3 Mb of non‐overlapping genome intervals (Table S2), in accordance with previous estimates computed using the gene‐space assembly of barley (Mascher *et al.*, 2013). For computing sequence coverage, only properly mapped paired‐ends (PE) reads were considered and on average 24.6 M PE per sample were counted, leading to an average sequencing depth of 40× over the 170 725 captured sequences. Analysis of the average per‐target coverage computed across the panel of 397 accessions indicated that 80% of captured sequences show a sequencing depth larger than 5×, which ensured sufficient coverage for subsequent analyses.

For each sample, properly mapped PE reads were counted within the genome coordinates of the 170 725 capture sequences. The resulting read count data were fitted in a beta‐binomial model and used to build optimized reference sets for detecting CNVs using ExomeDepth (Plagnol *et al.*, 2012). Because current algorithms for detecting CNVs based on read count data are prone to output results with unsatisfactory levels of type I error (Tan *et al.*, 2014), additional procedures were adopted to increase the confidence of genetic variant calling. First, an average per‐target analysis was carried out to remove sites with coverage below 5× because, with this sequencing depth, it is challenging to distinguish biases introduced with sequence capture from actual duplications and deletions. The output read count matrix was subsequently used for detecting CNVs. These were categorized based on whether they exhibited a significantly higher or lower number of reads than expected. Because our pipeline cannot reliably quantify the number of copies relative to the reference sequence, we collectively refer to these genetic variant groups as duplications or deletions, respectively. Second, duplications and deletions detected in less than three barley accessions were discarded. Overall, this procedure allowed us to call 1 037 381 duplications and deletions over the whole panel of 397 accessions and unveiled that 17.6% of the 170 725 captured sequences exhibit changes in copy number. Because captured targets are exons, contiguous duplications or deletions detected in each sample were merged and 197 407 CNV calls were inferred (Table S3). These were then mapped to 16 605 physical positions (CNV sites) across the seven barley chromosomes (Table S3). On average, 497 CNVs per barley sample were detected.

**Figure 1:**
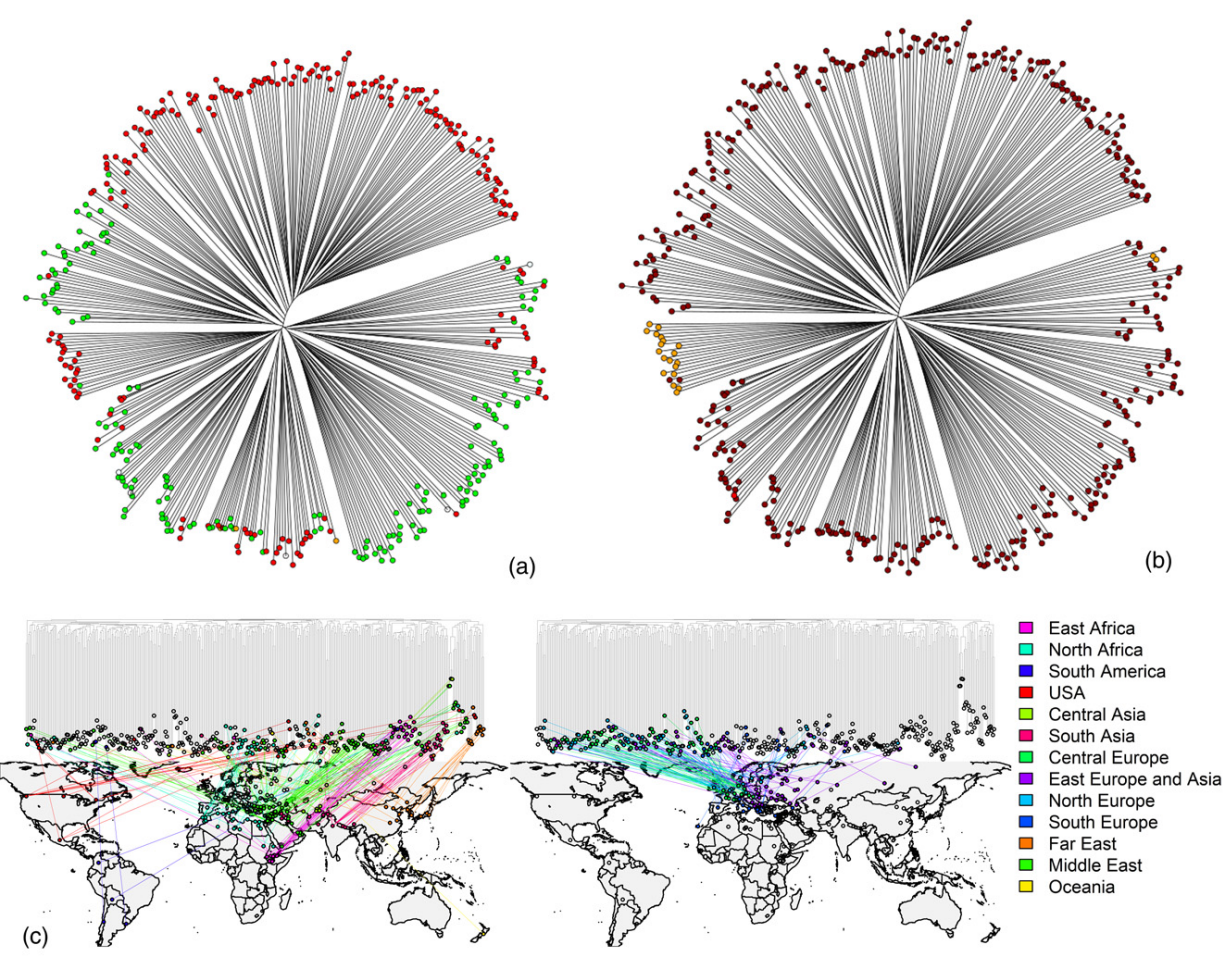
Copy number variant (CNV)‐based phylogeny of the 397 barley accessions. (a) In this phylogeny, two‐row and six‐row barley accessions are depicted in red and green, respectively. Accessions exhibiting mutant phenotypes for spikelet formation (*Hordeum vulgare* L. convar. *deficiens, Hordeum vulgare* L. convar. *intermedium* and *Hordeum vulgare* L. convar. *labile*) are depicted in yellow, white and brown, respectively. (b) In this phylogeny, domesticated barley accessions (*Hordeum vulgare* subsp. *vulgare*) and wild relatives (*Hordeum vulgare* subsp. *spontaneum* and feral *Hordeum vulgare* subsp. *agriocrithon*) are depicted in brown, orange and red, respectively. (c) Projection of the CNV‐based phylogeny onto a world map according to the geographic origin of barley accessions.

A two‐pronged strategy was pursued to assess the reliability of our CNV calling pipeline and estimate the residual type I error. As a first step, a CNV‐based phylogeny of the 397 barley accessions was computed using neighbor‐joining method and Euclidean distance (Figure 1). The resulting phylogeny showed separate clusters of two‐row and six‐row accessions (Figure 1a) and of wild and domesticated accessions (Figure 1b), reflecting the history of empirical breeding and selection of the genetic material. Similarly, the projection of tree tips onto a world map showed that the barley accessions investigated in the present study cluster according to their geographic origin (Figure 1c), demonstrating that our CNV phylogeny was consistent with that obtained using SNPs (Bustos‐Korts *et al.*, 2019).

**Figure 2:**
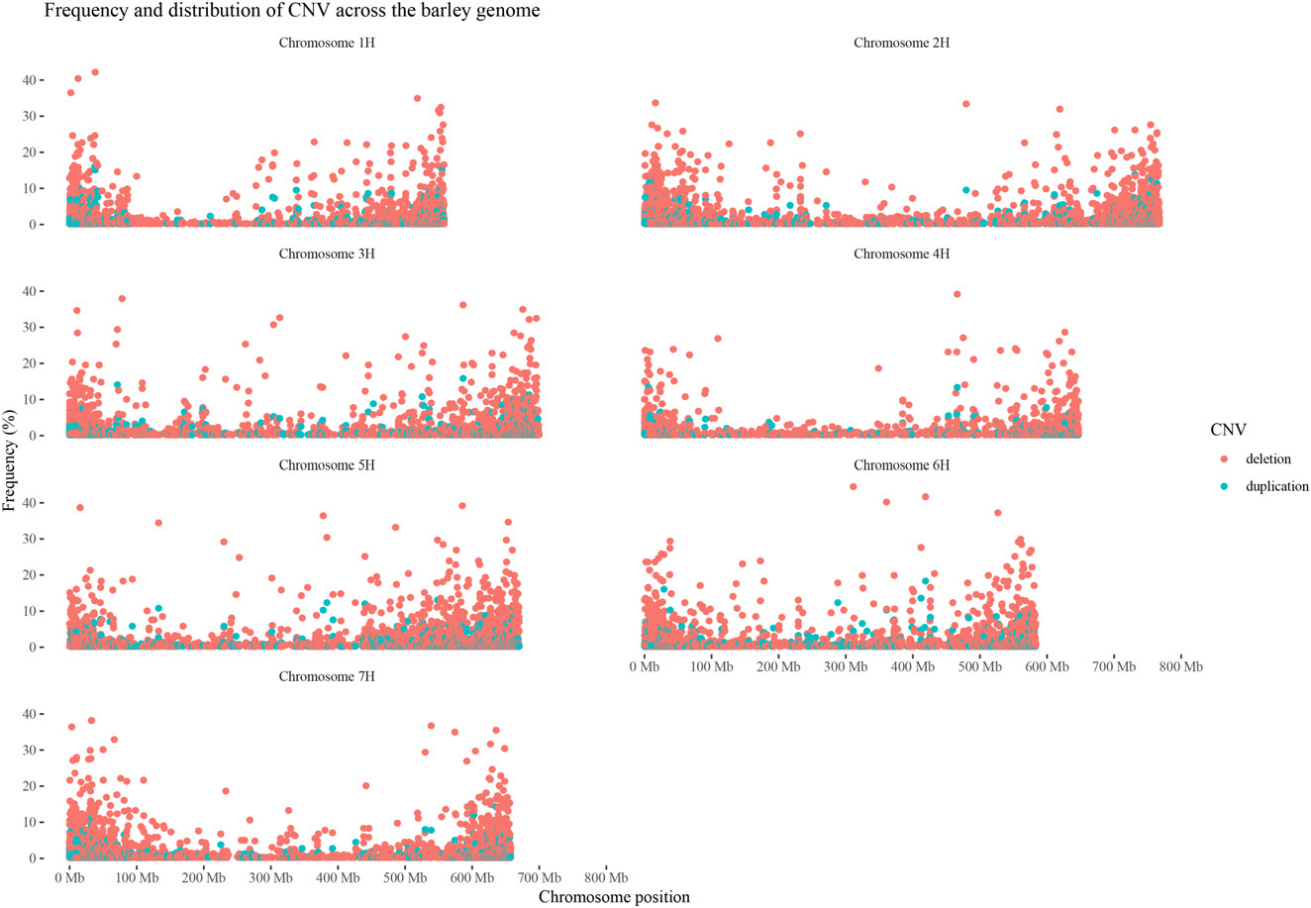
Distribution and frequency of copy number variant (CNVs) detected across the seven barley chromosomes. Plots show the genome coordinates of CNVs along the seven barley chromosomes (*x*‐axis), whereas the frequency (%) of each CNV in the panel of 397 accessions is reported on the *y*‐axis. Red and blue points of the plots indicate deletions and duplications, respectively.

The non‐stochastic clustering of barley accessions in the CNV‐based phylogeny indicated that CNV detection based on read count data generated reliable calls. To further assess the level of type I error, we selected 37 random CNVs, which were subsequently tested by a polymerase chain reaction (PCR) in 150 of the genotypes using primer pairs designed to target detected duplications and deletions (Table S4). For these 37 CNVs, structural changes were correctly identified in 142 out 150 samples (96.6%), demonstrating that CNVs were reliably identified. A very large fraction of the detected CNVs was present in the population at low frequency, although some deletions had a frequency higher than 40% across the whole panel of accessions (Figure 2).

On average, using the barley cv ‘Morex’ reference sequence, the deletions affecting barley gene content were estimated to be 3.81‐fold relative to the duplications, spanning from a minimum value of 3.45 of chromosome 1H to a maximum value of 4.20 of chromosome 4H (Table 1).

**Table 1:** Distribution of copy number variants (CNVs) across the seven barley chromosomes

Chromosome	Total number of CNVs	Number of deletions	Number of duplications	Deletion/duplication ratio
Chromosome 1H	2558	1983	575	3.45
Chromosome 2H	2941	2355	586	4.02
Chromosome 3H	2496	2001	495	4.04
Chromosome 4H	968	782	186	4.20
Chromosome 5H	2498	1973	525	3.76
Chromosome 6H	2104	1663	441	3.77
Chromosome 7H	3040	2393	647	3.70
All chromosomes	16 605	13 150	3455	3.81

To assess whether specific barley chromosomes are preferentially enriched in CNVs, the raw number of duplications and deletions detected in each chromosome was normalized relative to the length of per‐chromosome captured sequences (Table S2). The density of CNVs, measured as number of deletions or duplications per Mb of captured sequences, was computed to highlight the different incidence of CNV frequency across the coding sequences of barley chromosomes (Table 2). The density of deletions showed large variations. In chromosome 1H, 256.04 deletions per Mb of captured sequences were computed, whereas, in chromosome 4H, the deletion density was 102.62 (Table 2). A similar trend was observed for duplication densities: in chromosome 1H, 74.24 duplications per Mb were computed, whereas chromosome 4H showed a paucity of CNVs, with 24.41 duplications per Mb (Table 2).

**Table 2:** Distribution of copy number variants affecting coding sequences across the seven barley chromosomes

Chromosome	Density of deletions^a^	Density of duplications^b^
Chromosome 1H	256.04	74.24
Chromosome 2H	238.03	59.23
Chromosome 3H	204.94	50.70
Chromosome 4H	102.62	24.41
Chromosome 5H	200.75	53.42
Chromosome 6H	229.19	60.78
Chromosome 7H	260.11	70.33
All chromosomes	213.10	56.16

^a^ Number of deletions per Mb of per‐chromosome captured targets.

^b^ Number of duplications per Mb of per‐chromosome captured targets.

To test whether the low rate of CNV density observed in chromosome 4H departs significantly from the rates of other chromosomes, CNV densities were modelled as Poisson distributions and tested to assess whether pairs of CNV densities were different. *P* values of the pairwise Poisson’s tests revealed that CNV densities were significantly different and that the rate for chromosome 4H was significantly lower than that of the remaining barley chromosomes (Table 3).

**Table 3:** *P* values of pairwise Poisson's tests for comparing the rates of copy number variant (CNV) densities in barley chromosomes

	Chromosome 1H	Chromosome 2H	Chromosome 3H	Chromosome 4H	Chromosome 5H	Chromosome 6H
Chromosome 2H	0.36	–				
Chromosome 3H	5.05* × 10^−11^ *	1.76 × 10^−05^ *	–			
Chromosome 4H	1.33 × 10^−113^ *	1.97 × 10^−102^ *	3.36 × 10^−64^ *	–		
Chromosome 5H	4.89 × 10^−13^ *	5.00 × 10^−07^ *	1	5.35 × 10^−60^ *	–	
Chromosome 6H	0.02*	1	1.64 × 10^−2^ *	7.77 × 10^−81^ *	0.15*	–
Chromosome 7H	1	0.04*	6.51 × 10^−14^ *	5.09 × 10^−127^ *	2.90 × 10^−16^ *	0.15

a *Significant *P* values are marked with asterisks.

**Figure 3:**
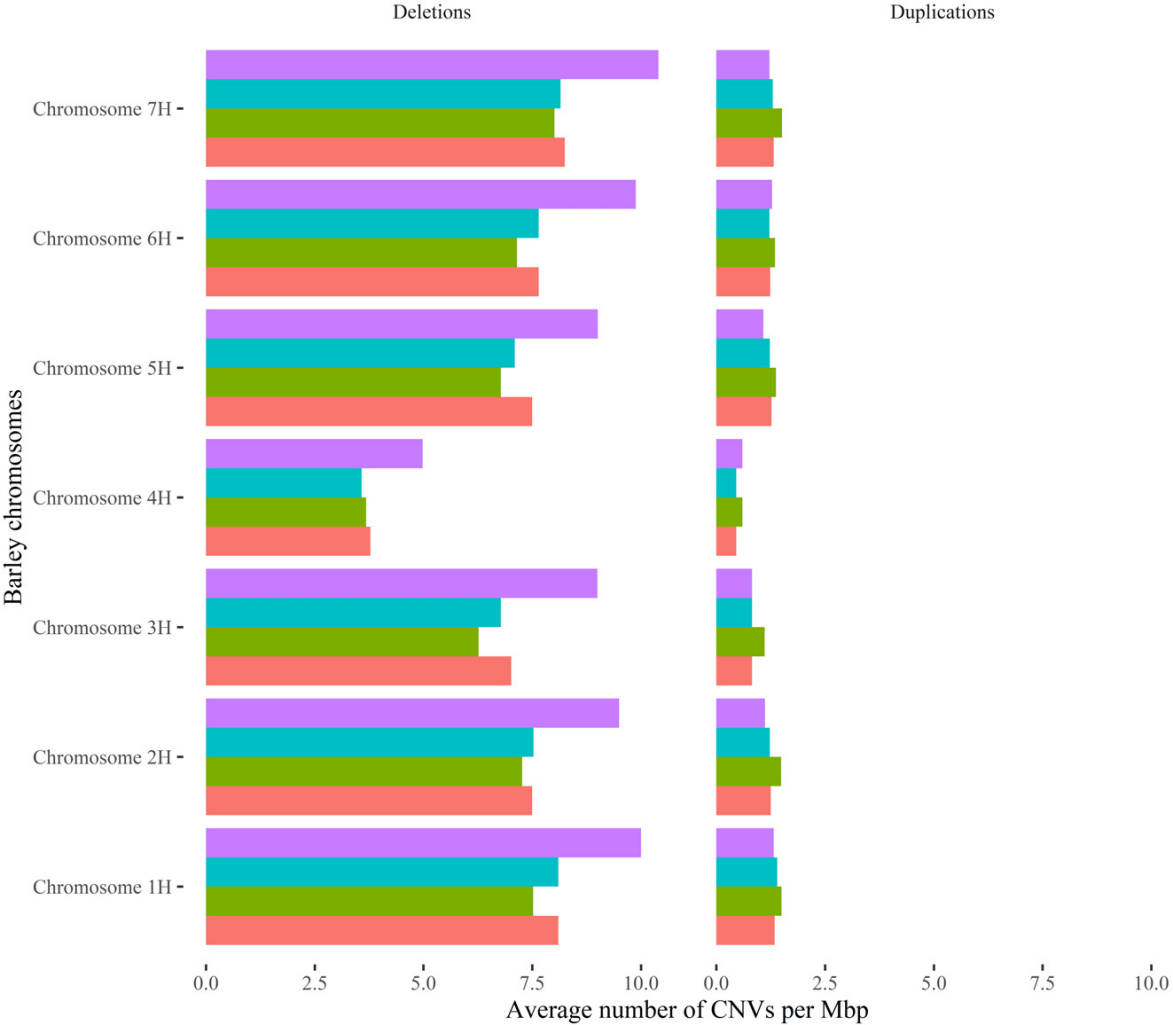
Average of per chromosome copy number variant (CNV) density computed in different categories of barley accessions. Bars report the average density of deletions (left bar plot) and duplications (right bar plot) detected in wild relatives (violet bars), landraces (light blue bars), cultivars (green bars) and in the whole panel of accessions (red bars).

The average density of CNVs affecting gene content across all accessions, cultivars and landraces showed that barley wild relatives, and to certain extent landraces, contain a significantly larger fraction of the deletion diversity compared to the cultivars, with this trend also being observed in all barley chromosomes (Figure 3). Conversely, the pattern of duplication densities across all barley chromosomes does not show statistically significant differences in landraces and cultivars (Figure 3).

### Functional impact of CNVs affecting barley gene content

**Figure 4:**
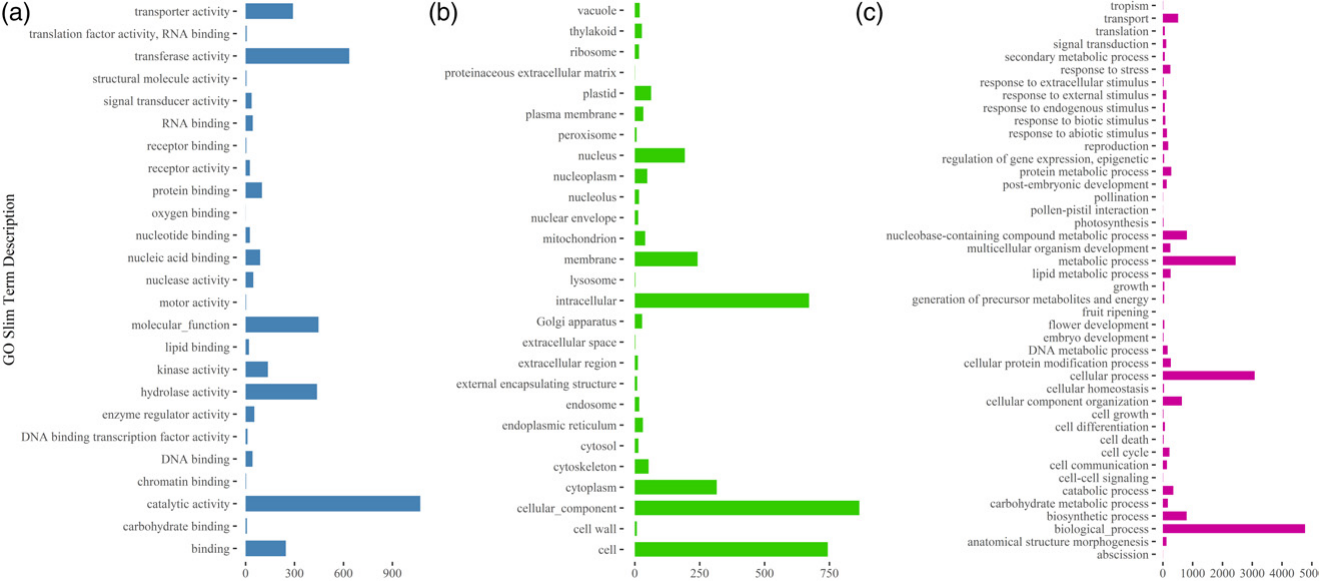
Overview of the ontology content of duplicated and deleted genes. Bars show the description of Gene Ontology (GO) Slim Term (*y*‐axis) of duplicated and deleted genes, whereas the count of each GO Slim term is reported on the *x*‐axis. (a) In this bar plot, the count of high‐level GO terms of ‘Molecular Function’ domain are reported, whereas, in (b) and (c), the count of high‐level GO terms of ‘Cellular Component’ and ‘Biological Process’ domains are reported, respectively.

To obtain insight into the biological and evolutionary implications of CNVs, the whole set of sequences used for designing exome capture probes was annotated using Gene Ontology (GO) terms. Using a homology‐based approach (Conesa and Gotz, 2008), 155 235 out 287 462 sequences (approximately 54%) used for designing exome capture probes were annotated with GO terms (Mascher *et al.*, 2013). The GO terms of this set of 155 235 sequences were subsequently associated with the barley genes in which captured sequences were unambiguously mapped. With this approach, CNVs were annotated with 4985, 927 and 2679 GO terms of the three domains ‘biological process’, ‘cellular component’, and ‘molecular function’, respectively. Categorization of these GO terms using the high‐level summary of functions implemented in the GO Slim terms (McCarthy *et al.*, 2006) showed that a large fraction of genes exhibiting changes in copy number is involved in transporter, transferase and hydrolase activities (Figure 4a). Moreover, examination of GO Slim terms indicated that genes showing changes in copy number are involved in shaping cellular and membrane components (Figure 4b; see also Table S5) and in metabolic and cellular processes (Figure 4c; see also Table S5).

**Figure 5:**
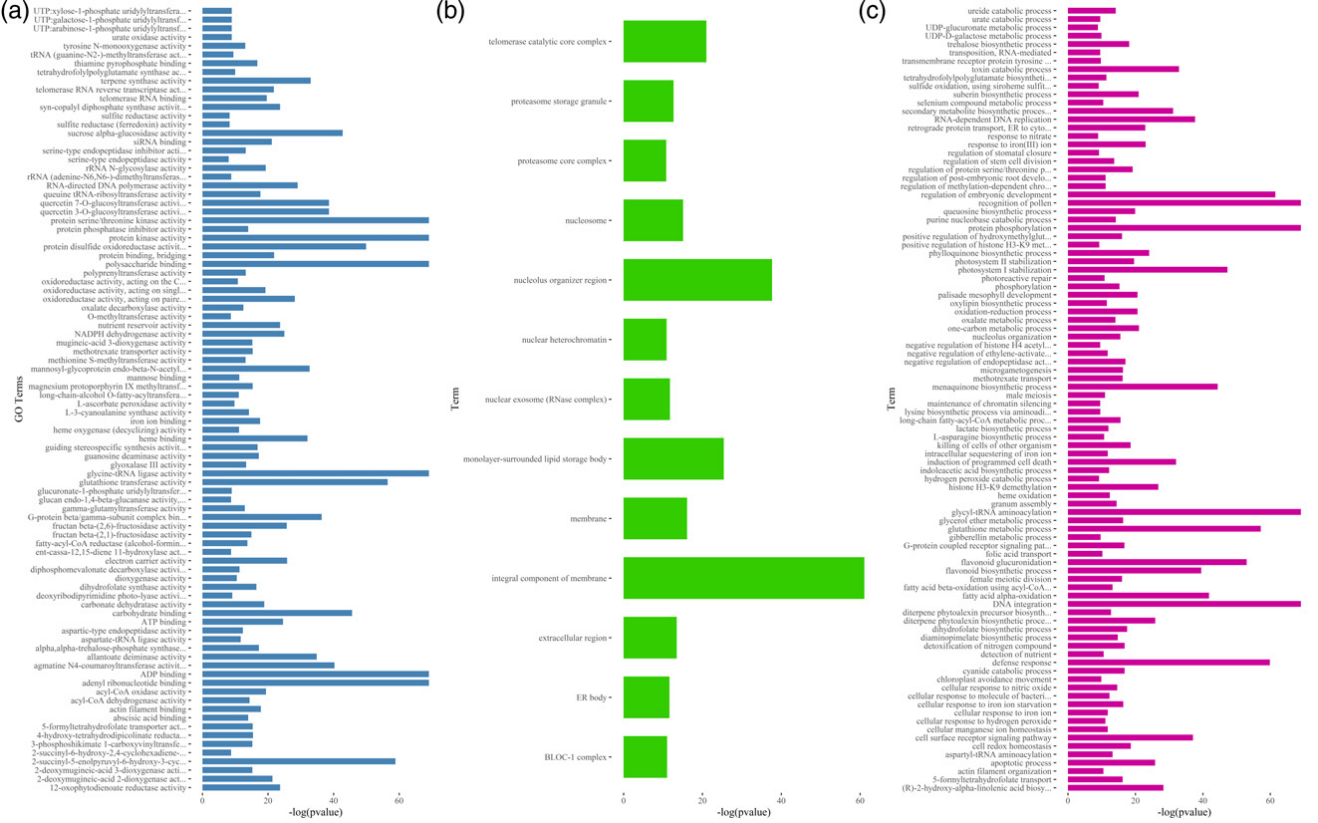
Gene Ontology (GO) enrichment in duplicated and deleted genes. The 193 GO terms (*y*‐axis) (FDR threshold ≤ 0.01) over‐represented in duplicated and deleted genes are plotted along the corresponding negative logarithm of their Fisher's *P* value (*x*‐axis). (a) Over‐represented GO terms of the ‘Molecular Function’, (b) ‘Cellular Component’ and (c) ‘Biological Process’ domains are reported, respectively.

To assess the incidence of over‐represented GO terms in duplicated and deleted genes, a GO enrichment analysis was carried out considering the whole set of barley genes for which the GO annotation was retrieved. Considering a false discovery rate (FDR) threshold of 0.01, computed using Benjamini–Hochberg procedure (Benjamini and Hochberg, 1995), 193 GO terms were found over‐represented in the set of duplicated and deleted genes (Figure 5; see also Table S6). GO enrichment analysis showed that genes with kinase, polysaccharide binding and ADP binding functions are more prone to be duplicated or deleted in barley (Figure 5a). Similarly, in duplicated and deleted genes, the enrichment analysis uncovered GO terms of the ‘Cellular Component’ domain related to ‘integral component of membrane’ (Figure 5b). Over‐represented GO terms of the ‘Biological Process’ domain and related to functions involved in the defense response, DNA integration and protein phosphorylation were also identified in genes showing copy number changes (Figure 5c; see also Table S6).

**Figure 6:**
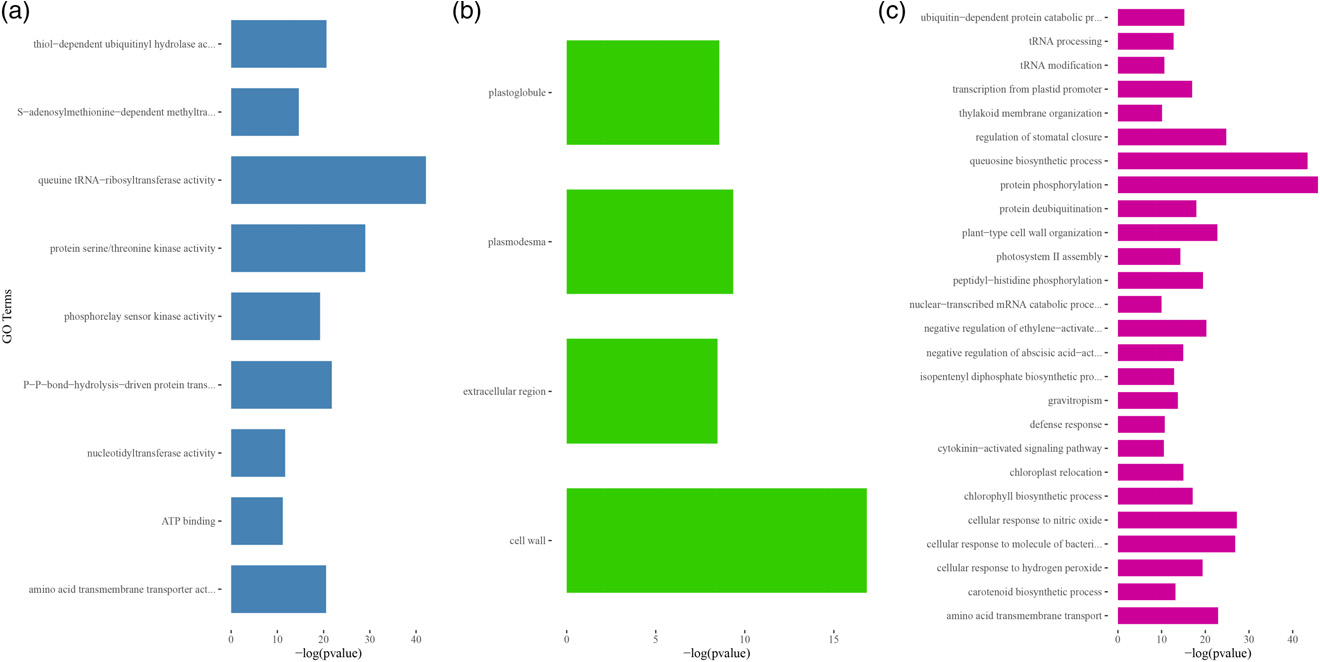
Gene Ontology (GO) enrichment of duplicated and deleted genes differentially detected in wild and domesticated accessions. The 39 GO terms (*y*‐axis) (FDR threshold ≤ 0.01) over‐represented in duplicated and deleted genes of wild accessions are plotted along the corresponding negative logarithm of their Fisher's *P* value (*x*‐axis). (a) Over‐represented GO terms of the ‘Molecular Function’, (b) ‘Cellular Component’ and (c) ‘Biological Process’ domains are reported, respectively.

Similarly, a GO enrichment analysis was carried out considering the set of duplicated and deleted genes that were detected exclusively in wild accessions to assess the functional categories of genes exhibiting CNVs that were lost during domestication (Figure 6). This analysis showed that the reduction of CNV diversity during the domestication process lead to the loss of CNVs affecting genes involved in queuine tRNA‐ribosyl‐transferase and protein kinase activity (Figure 6a), as well as in cell wall components (Figure 6b). Over‐represented GO terms of the ‘Biological Process’ domain and related to functional categories involved in protein phosphorylation, regulation of stomatal closure and cellular response to nitric oxide were also identified (Figure 6c).

### Revisiting of earlier reported CNVs using the barley reference sequence

The extent of barley gene CNVs was previously investigated in a limited panel of domesticated and wild accessions using the gene space assembly (International Barley Genome Sequencing Consortium *et al*., 2012), along with comparative genome hybridization (CGH) technology (Muñoz‐Amatriaín *et al.*, 2013). These data were revisited in light of the barley reference sequence to lift over the genome coordinates of earlier reported structural variants, which were subsequently compared with the pattern of gene CNVs detected with exome sequencing (ES) in the present study.

As a first step, the whole set of 115 003 contigs used for designing CGH probes (Muñoz‐Amatriaín *et al.*, 2013) was mapped against the reference sequence (Mascher *et al.*, 2017) and the mapping positions of these contigs were compared along the genome coordinates of ES targeted sequences. Overall, CGH probes target 228 603 non‐overlapping chromosome intervals and 46.04 Mb of the barley reference sequence compared to the 170 725 chromosome intervals and 61.3 Mb of ES probes. The CGH and ES targeted regions overlap for 46 814 chromosome intervals, which span 6.33 out 61.3 Mb (10.3%) of sequences analysed with exome capture technology: although ES and CGH probes were designed using two similar sets of contig sequences, CGH probes cover a small subset of the sequence captured with ES.

Because the panel of accessions analysed with ES does not include the whole set of genetic material analysed with CGH (Muñoz‐Amatriaín *et al.*, 2013), the comparison of CNVs detected with these two technologies was limited to sites in which deletions and duplications were identified. Overall, 8588 out 33 653 CNV sites identified with CGH and lifted over the barley reference sequence overlap or partially overlap with the 16 605 CNV sites identified with ES (Figure 1). The same comparison carried out with the unfiltered dataset of CNV detected with ES revealed that 13 369 overlapping structural variant sites were identified with both technologies (Figure 2). Although the use of different panels of genotypes limits this comparison, the analysis showed that a large fraction of CNV sites detected with ES was previously identified with CGH technology.

### Identification and nature of SDs in the barley genome

Identification of SDs in the reference sequence of barley cv ‘Morex’ (Mascher *et al.*, 2017) was pursued adopting a methodology based on sequence similarity search of high complexity regions. After masking interspersed repeats and low complexity regions of the reference sequence using the curated annotation of barley repetitive elements (Wicker *et al.*, 2017), the reference sequence was aligned against itself using chunks of 250 kb as queries to identify high similarity regions. Subsequently, data were parsed to exclude alignment pairs of query sequences matched against themselves and alignments shorter than 1 Kb.

**Figure 7:**
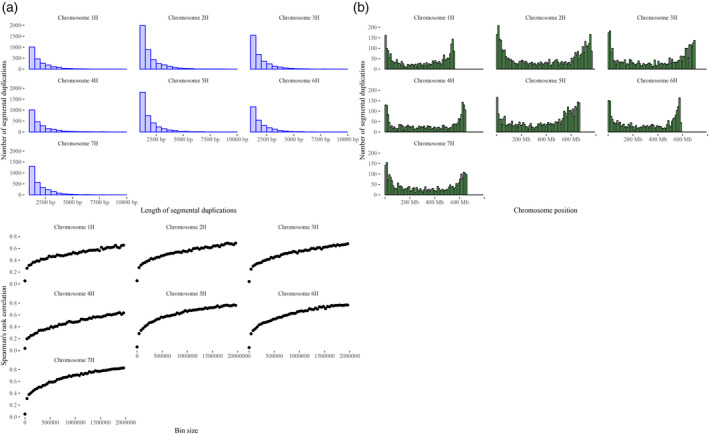
Frequency and length spectra of segmental duplications (SDs) and correlation with copy number variants (CNVs). (a) Length spectrum of SDs detected in barley cv ‘Morex’. (b) Histograms of SD distribution across the seven barley chromosomes. (c) For each of the seven plots, on the *y*‐axes, the values of Spearman rank correlation coefficient between SDs and CNVs are plotted, whereas, on the *x*‐axes, the values of bin size utilized for computing the Spearman rank correlation coefficient are reported. Only statistically significant values of Spearman rank correlation coefficient with *P* values lower that 0.001 are plotted.

Considering stretches of high complexity repeats with at least 95% identity, 20 853 SDs were identified across the seven barley chromosomes, which encompass approximately 40.6 Mb and cover 0.89% of the genome size. The length distribution (Figure 7a) showed that SDs spanning from 1 kb to 2kb are the most abundant in all chromosomes, whereas chromosomes 2H and 5H are the most SD‐rich (Figure 7a).

Among these SDs, 12 631 and 9114 have nucleotide identity of 98% and 99%, respectively and represent a subset of SDs that were recently fixed in the barley reference sequence (Table 4).

**Table 4:** Number of segmental duplications (SDs) identified in the reference sequence of barley cv ‘Morex’ using different identity thresholds

Number of SDs	Identity (%)	Length (bp)
20 853	> 95	> 1000
18 873	> 96	> 1000
16 107	> 97	> 1000
12 631	> 98	> 1000
9114	> 99	> 1000

The density of SDs indicated that the ends of chromosome arms contain more SDs and this trend was observed for all chromosomes (Figure 7b). To unlock the nature of these SDs, their genomic coordinates were compared with the high and low confidence annotations of barley: 5743 out 20 853 SDs fully or partially overlap high confidence genes, whereas the remaining SDs are not part of the high confidence annotated gene content. Considering the low confidence annotation (Mascher *et al.*, 2017), 2714 out 20 853 SDs overlap chromosome intervals in which genes with annotation of unknown function or without functional annotation were detected (Mascher *et al.*, 2017). These findings reflect previous estimates highlighting a large fraction of barley genes as originating from duplication events that shaped gene families with multiple members (Mascher *et al.*, 2017).

**Figure 8:**
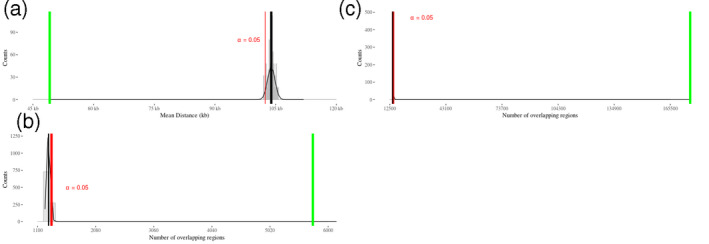
Association analysis of segmental duplications (SDs) based on permutation tests. In all plots, the measured value (green line) and the expected value (black line) obtained after the randomization of sequence intervals are reported. (a) In this plot, the average distance of SDs (*x*‐axis) with their closest genes was compared with the lower bound of the expected average distance (red vertical line); (b) In this plot, the number of overlaps (*x*‐axis) between SDs and annotated genes was compared with the upper bound (red line) of the expected number of overlaps in case of random distribution. (c) In this plot, the number of overlaps (*x*‐axis) between SDs and copy number variant (CNV) sites was compared with the upper bound (red line) of the expected number of overlaps.

Because the distribution of SDs in barley chromosomes (Figure 7b) shows the same pattern of the predicted coding sequences (Mascher *et al.*, 2017), an association analysis between these genomic regions was carried out based on permutation tests to determine whether SDs overlap predicted coding regions more than expected. The average distance of SDs with their closest gene is 47 kb (Figure 8a, green vertical line), whereas the expected lower bound of the average distance under a random distribution of genomic features is approximately 105 kb (Figure 8a, red vertical line), corroborating the finding that SDs and genes are strictly associated in the barley genome. The analysis revealed that SDs and predicted coding sequences are strictly associated because the 5743 overlaps between these genomic regions (Figure 8b, green vertical line) are significantly higher than the upper bound of expected overlaps under a random distribution (Figure 8b, red vertical line).

### CNVs co‐occur with SDs identified in the barley reference sequence

Pioneering studies on structure and function of the human genome demonstrated that CNV abundance increases in SD‐rich sequence intervals, and SD‐mediated NAHR was suggested as a possible mechanism of CNV formation (Freeman *et al*., 2006; Goidts *et al.*, 2006; Perry *et al.*, 2006). To assess whether SDs are hot spots for the formation of CNVs in barley, Spearman rank correlation coefficients were computed between the SDs and the CNVs detected in the panel of 397 accessions. SDs were binned into increasing sequence intervals (from 40 kb to 2 Mb) and their associations with the number of CNVs detected in the panel of 397 accessions and mapped within the same bins were examined, computing Spearman rank correlation coefficients between these two structural features.

The values of Spearman rank correlation coefficients were finally computed as a function of bin sizes (Figure 7c), which show high and statistically significant correlations between SDs and CNVs when bin sizes equal or larger than 1.5 Mb are used for computation (rank correlation higher than 0.7) (Figure 7c). These high values of rank correlation imply that a monotonic function ties SDs and CNVs and that SD‐rich sequence intervals of the reference sequence are those regions that are more prone to gain extra copies or lose DNA sequences. Similarly, an association analysis of the sites where CNVs were detected with SDs was carried out to determine whether CNV formation is associated with the closeness of SDs. The results of the association analysis clearly show that CNV sites overlap SDs more than expected under a random distribution (Figure 8c), demonstrating that the presence of CNVs is statistically associated with the closeness of SDs.

## DISCUSSION

In the present study, we used a sequence‐based approach that relies on read count data generated with ES to unveil changes in the copy number of barley genes. Considering the large number of accessions and the type of genetic material examined, to date, the present study has delivered the most comprehensive overview of CNVs that affect gene content in cultivars, landraces and wild relatives of barley.

Beyond SNP identification, ES was extensively applied for seeking somatic and germline CNVs in human species. This practice showed that methodologies for CNV detection based on read count might output results that are error‐prone because of the unsatisfactory FDR (Tan *et al.*, 2014). Currently, several algorithms have been proposed for detecting CNVs using read count data generated with ES to examine genomic aberrations of human individuals, although there is evidence that new statistical paradigms are needed to improve accuracy and sensitivity (Zare *et al.*, 2017). On the other hand, in plants, exome capture and sequencing represent groundbreaking technologies for detecting genome‐wide DNA variants at the same time as maintaining acceptable costs (Warr *et al.*, 2015). In the present study, we implemented several strategies to reduce the FDR of our CNV detection procedure as much as possible and we used clustering analyses and targeted amplifications to determine the performance of our procedure. Along with the molecular analyses conducted for validating a subset of duplications and deletions, the CNV‐based phylogeny proved that the structural changes identified in the present study correctly cluster barley accessions based on their row type (six‐row and two‐row) and category (domesticated and wild relatives), corroborating the high quality and performance of our CNV detection strategy.

### CNVs contribute to shape barley genome diversity

Along with other structural changes, CNVs were proposed to underlie the speciation of humans from other non‐human primates (Perry *et al.*, 2006; Kim *et al.*, 2008; Girirajan *et al*., 2011), which would have led to substantial genome re‐arrangements that allow the acquisition of new functions, whereas, in plants, there is evidence that changes in the copy number of genes are pervasive in certain crops and constitute the genetic bases of important agronomic traits (Sutton *et al.*, 2007; Swanson‐wagner *et al.*, 2010). In the present study, we surveyed genome‐wide CNVs affecting gene content in a panel of barley accessions including 172 cultivars, 171 landraces and 22 wild relatives. Previous studies using gene re‐sequencing and amplified fragment length polymorphism technology (Vos *et al.*, 1995) uncovered a loss of diversity in cultivars compared to landraces and wild relatives (Kilian *et al.*, 2006, 2007; Condón *et al.*, 2009; Fricano *et al.*, 2009). Leveraging the CNVs detected in the present study, a reduction of deletions was observed in cultivars and in landraces compared to wild accessions, whereas the same pattern was not observed for duplications (Figure 3). Similarly, our analysis pointed out a slight reduction of CNV diversity in barley cultivars compared to landraces (Figure 3). Although the reduction of deletions can be explained considering that barley domestication and breeding narrowed the genetic diversity in the domesticated accessions (Kilian *et al.*, 2006), the pattern of duplications in cultivars and landraces (Figure 3) suggest that newly duplicated sequences would rapidly diverge, accumulating point mutations that mask their formation and our ability to detect these events using exome capture and sequencing.

The results reported in the present study limit our conclusions regarding CNVs that affect gene content and, consequently, the actual number of deletions and duplications that segregate in our accessions could be underestimated. Moreover, the current availability of a single reference sequence of barley cv ‘Morex’ contributes to shrinking our capability of determining CNVs of sequences that are not present in this reference.

### CNVs are pervasive across barley gene content

Considering the whole panel of 397 diverse accessions of barley, the ES‐based pipeline used for detecting CNVs unveiled that 17.6% of the 170 725 captured sequences exhibit changes in copy number. Because captured targets represent gene exons in most cases, contiguous deletions or duplications were merged and 16 605 CNV sites were inferred.

These 16 605 CNV sites represent an estimate of DNA segments that can be duplicated or deleted in barley and their intersection with annotated gene models indicates that this genome can bear losses or extra copies of sequences in approximately 10% of predicted genes. This value is comparable to the findings obtained applying comparative genomic hybridization (CGH) technology on a limited set of accessions using the gene space assembly of barley (Muñoz‐Amatriaín *et al.*, 2013). CNV studies carried out in a panel of domesticated maize accessions and teosinte lines showed that more than 10% of the genes annotated in the B73 reference genome exhibit CNVs (Swanson‐wagner *et al.*, 2010). Similarly, our findings show evidence that the fraction of genes exhibiting changes in copy number in barley and maize is comparable.

The loss of gene copies found in barley would be explained by the high level of gene families with multiple members annotated in this species (Mascher *et al.*, 2017). It is plausible that genes belonging to the same gene family would have redundant or partially redundant functions, which in turn compensate for possible deleterious effects of losses of gene copies. In barley, there are notorious examples of genes that show CNVs among different accessions. For example, CNVs of *CBF* genes at *Fr‐H2* locus were reported in barley cultivars using a targeted approach based on gene copy quantification (Francia *et al.*, 2016). *CBF* genes underlie frost tolerance trait and their number of copies and paralogs was associated with the level of frost tolerance in barley and other cereals (Francia *et al.*, 2016; Sieber *et al.*, 2016). In the present study, CNVs of *CBFs* previously reported were detected in several barley accessions (Francia *et al.*, 2016) along with CNVs of *Vrn‐H1*, another important gene that has pleiotropic effects on frost tolerance. Moreover, the detection of duplications affecting gene content hints that these extra copies of DNA would play important roles for barley adaptation to different environmental conditions, as reported previously (Sutton *et al.*, 2007; Francia *et al.*, 2016).

Comparison of the density of deletions or duplications across different chromosomes showed that chromosome 4H contains a significantly lower number of CNVs, confirming the previous report that pointed out the depletion of CNVs in this chromosome using CGH technology (Muñoz‐Amatriaín *et al.*, 2013). Chromosome 4H would undergo a lower rate of events that lead to the formation of deletions and duplications as a result of either the lack of regions that promote instability or reduced meiotic recombination, as suggested previously (International Barley Genome Sequencing Consortium *et al*., 2012; Mascher *et al.*, 2017).

### Changes in the copy number of genes are associated with SD‐rich regions

The availability of a high‐quality reference sequence allowed us to unlock the extent and occurrence of SDs in the barley genome. A large fraction of newly formed SDs partially or fully overlap predicted genes in both high confidence and low confidence annotations, reflecting the high number of families with duplicated genes that were annotated in the barley genome (Mascher *et al.*, 2017). Although predicted genes explain a significant part of SDs identified, the nature of SDs that did not overlap with either annotated mobile elements or coding sequences is still unclear and could be explained by postulating the existence of other genes or pseudo‐genes that were not considered during the annotation process.

The findings reported in the present study demonstrate that CNVs are not randomly distributed across barley‐coding sequences, although they tend to occur in the SD‐rich regions identified in the barley reference sequence (Figure 7c). SDs overlap more than expected CNV sites, indicating that they would shape regions of genomic instability, which foster the emergence of new CNVs. The molecular mechanisms that generate CNVs were extensively described in yeast, *Drosophila melanogaster* and primates (Goidts *et al.*, 2006; Kim *et al.*, 2008; Salse *et al.*, 2008; Daines *et al.*, 2009; Conrad *et al.*, 2010; Zecevic *et al.*, 2010; Zhang *et al.*, 2013), although our understanding of their incidence in plant genomes is still limited. An obvious hypothesis is that, in barley, recent SDs offer adequate nucleotide identity for enabling the formation of new unbalanced structural changes via NAHR. The co‐occurrence of CNVs in SD‐rich regions is a signature of SD‐mediated CNV formation (Figure 7c) that was unveiled in the present study and hints that NAHR, similar to mammalian genomes, could shape CNVs affecting barley‐coding sequences, although other mechanisms were proposed.

Along with previous findings (Muñoz‐Amatriaín *et al.*, 2013), the present study has shown that, in the barley genome, deletions are approximately four‐fold more frequent than duplications. Although we cannot exclude the possibility that the divergence of newly duplicated sequences masks our ability to detect these events, it is plausible that the duplications and deletions occur at different rates in the barley genome, suggesting that NAHR mediated by SD pairs located in the same chromatids could be more frequent than NAHR mediated by SD pairs located in different chromatids (Chen *et al.*, 2014). Investigating the flanking regions of deletions and duplications, sequence signatures of CNV formation based on double‐strand break repair via single‐strand annealing were reported on 41.1% of CNVs of barley (Muñoz‐Amatriaín *et al.*, 2013). A possible reason for these apparently different findings is dependent on CGH, which was used for detecting CNVs in a small panel of 16 wild and domesticated barley accessions in a previous CNV study (Muñoz‐Amatriaín *et al.*, 2013). Because CGH does not allow the examination of sequences with high sequence similarity, CNVs in SD‐rich regions were probably not considered in the previous study (Muñoz‐Amatriaín *et al.*, 2013). The present study shows evidence of SD‐mediated formation of CNVs in barley, a mechanism that has been proposed several times in plants (Muñoz‐Amatriaín *et al.*, 2013; Bai *et al.*, 2016). Further studies on barley CNVs in non‐coding sequences are needed to explore the potential role of both NAHR‐based and double‐strand break‐based mechanisms in the formation of unbalanced structural changes in barley.

Overall, the landscape of the CNVs that have been revealed in the present study provides evidence for widespread changes in the copy number of genes, which in turn reflects the dynamic nature of the barley genome. Moreover, our findings pave the way for a better understanding of the gene content of core and dispensable genomes of this species for evolutionary studies (Morgante *et al*., 2007). As already demonstrated for frost and boron‐tolerance traits, it is likely that, along with SNPs, CNVs significantly contribute to barley phenotypic diversity, although further investigations are necessary to document the extent to which these structural variants affect other important traits. The use of CNVs in genome‐wide association studies would allow a better understanding how these structural variants underlie barley phenotypic variation and enable their exploitation for breeding.

We have demonstrated that changes in copy number of genes are widespread across the barley genome and that these structural variants contribute to shaping the genetic diversity of cultivars, landraces and wild relatives, affecting genes with specific functions. Moreover, we report that SD‐rich sequences are regions of the barley genomes in which CNV formation rate is higher than expected and speculate that molecular mechanisms based on similarity of SDs (e.g. NHAR) may be involved in changing copy number of genes. The list of CNVs identified in the present study comprises a new asset for understanding the genome biology and evolution of barley, as well as the genetic bases of complex traits.

## EXPERIMENTAL PROCEDURES

### Plant materials

The genetic material examined in the present study has been extensively described elsewhere (Bustos‐Korts *et al.*, 2019) and relevant information regarding the classification and the origin, type and of selected accessions is provided in Table S1. In brief, a panel of 397 out 403 barley accessions previously described (Bustos‐Korts *et al.*, 2019) was selected for the study, including 172 formally bred cultivars released in Europe, Asia and Americas, 171 landraces collected in Europe, Asia, Middle East and Africa, and 22 wild relatives of barley (*Hordeum spontaneum* subsp. *spontaneum* and *Hordeum spontaneum* subsp. *agriocrithon*) collected in Middle East areas. Another 32 domesticated accessions for which the categorization as cultivar or landrace was questionable were included and examined (Bustos‐Korts *et al.*, 2019).

### Preparation of exome capture library and sequencing

Genomic DNA (gDNA) was extracted from barley leaf material from a single plant for each genotype. DNA samples were checked with a Genomic DNA ScreenTape on an Agilent 2200 Tape Station System (Agilent, Santa Clara, CA, USA) to verify gDNA integrity. Samples were quantified using a Picogreen assay (Thermo Fisher, Walthem, MA, USA) and normalised to 20 ng µl^−1^ in 10 nm Tris‐Hcl (pH 8.0) as suggested in the NimbleGen SeqCap EZ Library SR protocol. The gDNA was fragmented to a size range of 180–200 bp using Covaris microTUBES and a Covaris S220 Instrument (Covaris, Woburn, MA, USA) and whole genome libraries were prepared in accordance with the Kapa Library Preparation protocol. Libraries were quantified using a Nanodrop spectrophotometer (Thermo Fisher) and analysed electrophoretically with an Agilent 2200 Tape Station System using a D1000 ScreenTape. Libraries were pooled in 8‐plex and used for the hybridization with the barley SeqCap Ez oligo pool (Design Name: 120426_Barley_BEC_D04) (Mascher *et al.*, 2013) in a thermocycler at 47°C for 48 h. Capture beads were used to pull down the complex of capture oligos and genomic DNA fragments and unbound fragments were removed by washing. Enriched fragments were amplified by PCR and the final library was quantified by quantitative PCR and visualised using the Agilent 2200 Tape Station. Sequencing libraries were normalised to 2 nm, and NaOH was denatured and used for cluster amplification on the cBot (Illumina, San Diego, CA, USA). The clustered flow cells were sequenced on Illumina HiSeq2000 with an 8‐plex strategy (i.e., 8 samples per HiSeq lane) with a 100 bp paired‐end run module.

### Analysis of whole ES data

Target regions utilized for designing exome capture probes (http://sequencing.roche.com/content/dam/rochesequence/worldwide/shared‐designs/barley_exome.zip) were mapped against the reference sequence of barley cv ‘Morex’ (Mascher *et al.*, 2017) with bwa‐mem 0.7.15 (Li and Durbin, 2009). Mapping positions of captured sequences were extracted from the BAM file of alignments and converted in BED format using bam2bed (Neph *et al.*, 2012). Subsequently overlapping BED records were collapsed using the merge command of bedops 2.4.20 (Neph *et al.*, 2012) to uncover the actual portions of the barley genome that are examined using barley whole exome capture.

Sequence quality control was assessed with FastQC (Andrews, 2010). Raw Illumina reads were then quality trimmed to a base quality of 20 from both ends with trimmomatic, version 0.30 (Bolger *et al*., 2014). Only correctly paired reads longer than 70 bp were used for further processing. Trimmed reads were then mapped to the reference genome with bwa, version 0.7.15, using the mem algorithm with default parameters (Li and Durbin, 2009). The resulting BAM files were sorted with samtools (http://www.htslib.org) (Li and Durbin, 2009) and duplicate reads were marked and removed with picard (Board Institute, 2016) using the ‘MarkDuplicates’ command. Coverage at each captured sequence was computed using samtools depth (Li, 2011) considering only properly mapped paired reads. Captured sequences exhibiting a coverage lower than 5× were removed from all subsequent analyses. The average sequencing coverage across the whole set of captured sequences was computed in the r statistical environment using rsubread, version 1.28 (Liao *et al*., 2013; R Developmental Core Team, 2015) including the count of PE fragments that overlap contiguous captured sequences. PE fragment counts obtained for each sample were subsequently merged in the r environment for creating a numeric matrix, which was subsequently utilized for detecting copy number variants.

### Detection of copy number variants and validation

Read count data were processed in the r statistical environment (R Developmental Core Team, 2015) with the r package ‘ExomeDepth’ for detecting CNVs (Plagnol *et al.*, 2012) setting the expected exon length at 1000 bp and the minimum quality mapping score at 30. CNVs detected in less than three barley accessions were discarded and not considered for validation. Contiguous deletions or duplications of captured sequences detected in the same accession were merged and the resulting CNVs were utilized for constructing a phylogeny based on the neighbor‐joining method and Euclidean distance utilizing the r packages ‘ape’ and ‘phytools’ in the r statistical environment (Saitou and Nei, 1987; Paradis *et al*., 2004; Revell, 2016).

### Identification of SDs in the barley reference sequence

For surveying the occurrence of SDs, all known repetitive elements of the barley reference sequence were masked utilizing the most recent and accurate annotation of transposable elements (Wicker *et al.*, 2017) and, subsequently, the masked chromosome sequences were split in chunks of 250 kb. These chunks were aligned against the masked reference sequence of barley for identifying homologous sequences using standalone blast, version 2.5.0 (https://blast.ncbi.nlm.nih.gov) (Altschul *et al.*, 1990; Camacho *et al.*, 2009). Alignment records obtained from blast analyses were subsequently parsed for identifying homologous sequence pairs sharing a nucleotide identity higher than 95% and larger than 1 kb using python, version 2.7.9 (https://www.python.org) along with the package ‘Biopython’ (Cock *et al.*, 2009). Alignment records were transformed in a BED file using custom python scripts and overlapping regions were subsequently collapsed using the bedops ‘merge’ command (Neph *et al.*, 2012).

### GO ontology and enrichment analysis

To explore the ontology content of duplicated and deleted genes, the whole set of 283 096 sequences used for designing exome capture probes was annotated with GO terms using blast2go (Conesa and Gotz, 2008). Subsequently, GO terms of these sequences were assigned to the genomic coordinates in which captured sequences were unambiguously mapped. The high‐level summary of functions implemented in GO Slim terms (McCarthy *et al.*, 2006) was used for summarizing the ontology content of duplicated and deleted genes.

Enrichment analysis was conducted in the r statistical environment using the r package ‘TopGO’ (Alexa *et al*., 2006; R Developmental Core Team, 2015) for identifying GO terms that were over‐represented and under‐represented in the set of duplicated and deleted genes and functional categories associated with set of duplicated and deleted genes that were lost in the domesticated accessions. For carrying out GO enrichment for the first analysis, the whole set of mapped sequences was utilized as baseline, whereas the over‐ and under‐represented GO terms were investigated in deleted and duplicated genes using the ‘elim’ algorithm implemented in ‘TopGO’ for selecting the most stringent subset of over‐represented and under‐represented GO terms. For identifying GO terms associated with duplicated and deleted genes that were lost during the domestication process, the whole set of mapped sequences was used as baseline, whereas the over‐ and under‐represented GO terms were investigated in deleted and duplicated genes that were detected exclusively in wild accessions, using the ‘elim’ algorithm implemented in ‘TopGO’.

The FDR threshold was calculated utilizing Benjamini–Hochberg procedure (Benjamini and Hochberg, 1995). Bar plots were generated using the package ‘ggplot2’ in the r statistical environment (R Developmental Core Team, 2015; Wickham, 2016).

### Association analysis of SDs with CNV sites and predicted genes

Histograms of SD distribution across barley chromosomes were computed in bins of 50 kb in the r statistical environment (R Developmental Core Team, 2015) parsing the BED file describing the genome coordinates of SDs with a nucleotide identity higher than 95%.

Association analyses between SDs and CNVs detected in the panel of barley accessions were computed using Spearman rank correlation coefficient, binning barley chromosomes in increasing intervals from 40 kb to 2 Mb. Within each interval, Spearman rank correlation coefficient was calculated in the r statistical environment (R Developmental Core Team, 2015) between the number of SDs unveiled in the reference sequence and the number of CNVs detected in the panel of 397 barley accessions. For assessing the non‐random association of SDs with CNV sites or predicted high confidence genes, 1000 permutation tests were carried out between pairs of features (SD and CNV sites; SD and predicted high confidence genes), randomizing features over the non‐masked space of each chromosome to compute the expected number of overlaps under the hypothesis of random distributions of these genomic features. Similarly, the expected average distance of SDs with the closest high confidence gene was computed permuting these genomic features over the non‐masked space of each chromosome 1000 times. The r package ‘regioneR’ (Gel *et al.*, 2016) was utilized for these computations and the results were plotted using the r package ‘ggplot2’ (Wickham, 2016).

## CONFLICT OF INTERESTS

The authors declare that they have no competing interests.

## AUTHOR CONTRIBUTIONS

AF, LR and LC conceived the study. AF led the study and carried out the data analysis, AF wrote the paper with significant contributions by RW, BK, LR and LC. BK, RW, JR, LR and LC assembled the panel of barley accessions. LR coordinated the exome sequencing of the barley collection. CF carried out library preparation, as well as capture and sequencing. GB carried out validation experiments and PB conducted GO annotations. All authors read and approved the final manuscript submitted for publication.

## Supporting information


**Table S1.** List of the 397 diverse barley accessions analysed in this study.
**Table S2.** Overall length of captured targets across the seven barley chromosomes.
**Table S3.** Whole list of CNV calls detected in the panel of 397 barley accessions.
**Table S4.** List of 37 primer pairs utilized for validating CNVs DELETE ONE PRIMER PAIR.
**Table S5.** Ontology content of gene CNVs.
**Table S6.** List of enriched GO terms.

## Data Availability

The raw sequencing data analyzed in this manuscript have been deposited in the European Nucleotide Archive under the study number: PRJEB33527.
